# Visceral obesity determined in routine preoperative CT scans predicts risk of postoperative burst abdomen

**DOI:** 10.1038/s41598-023-48714-0

**Published:** 2023-12-05

**Authors:** Matthias Mehdorn, Benedikt Schnarkowski, Yusef Moulla, Johanna Pape, Timm Denecke, Ines Gockel, Woubet Tefera Kassahun, Hans-Jonas Meyer

**Affiliations:** 1 Department of Visceral, Transplant, Thoracic and Vascular Surgery, University Hospital of Leipzig, Liebigstraße 20, 04103 Leipzig, Germany; 2 Department of Diagnostic and Interventional Radiology, University Hospital Leipzig, Leipzig, Germany; 3 Department of Pediatric Radiology, University Hospital Leipzig, Leipzig, Germany

**Keywords:** Risk factors, Comorbidities, Preventive medicine, Computed tomography, Colorectal surgery

## Abstract

Burst abdomen (BA) remains a severe postoperative complication after abdominal surgery. Obesity is a known risk factor for postoperative complications but objective parameters such as body mass index fail to predict BA after abdominal surgery. In recent literature, CT-derived body composition assessment could predict obesity-related diseases and surgical site infections. We report data from the institutional wound register, comparing patients with BA to a subgroup of patients without BA. The CT images were evaluated for intraabdominal and subcutaneous fat tissues. Univariate and multivariate risk factor analysis was performed in order to evaluate CT-derived obesity parameters as risk factor for BA. 92 patients with BA were compared to 32 controls. Patients with BA had significantly more visceral obesity (VO; *p* < 0.001) but less subcutaneous obesity (SCO) on CT scans. VO and SCO both were positively correlated with BMI (r = 0.452 and 0.572) but VO and SCO were inversely correlated (r = −0.189). Multivariate analysis revealed VO as significant risk factor for postoperative BA (OR 1.257; 95% CI 1.084–1.459; *p* = 0.003). Our analysis of patients with postoperative BA revealed VO as major risk factor for postoperative BA. Thus, preoperative CT scans gives valuable information on possible risk stratification.

## Introduction

Burst abdomen (BA), also known as abdominal wall dehiscence, is an acute complication after abdominal surgery that is characterized by the breakdown of all abdominal wall layers. The incidences of BA range between 1 and 6.6% in the literature^1,2^ depending on the operative scenario and the individual patient’s risk profile. To prevent any possible progression of evisceration, urgent closure of the abdominal wall will be mandatory. Subsequently, patients are at high risk of developing an incisional hernia^3^.

Previous studies showed surgical site infection (SSI) as major risk factor for BA^4,5^. This may be due to an inflammatory affection of the abdominal wall with consecutive breakdown of the abdominal wall integrity at the site of incision. Furthermore, poor nutritional status^5^, chronic steroid use^5^, chronic pulmonary disease and diabetes mellitus^4^ have also been identified as relevant prognostic factors.

In a previous study, we characterized risk factors for BA in patients with superficial surgical site infections after abdominal surgery being liver cirrhosis, intestinal resection and emergency surgery^6^. Additionally, we revealed postoperative delirium as risk factor for primary and recurrent BA.

Recently, body composition assessment by imaging modalities is of great importance. The aim is to define additional measurements in established imaging modalities to gain further information from the acquired image data sets. Several studies have linked abdominal adipose tissue to SSI with different measurements being effective predictors of postoperative SSI: median subcutaneous fat in abdominal surgery^7^, high visceral-to-subcutaneous-fat ratio^8^ or visceral fat area alone both in gastric cancer surgery^9^. However, there is no standardized measurement to predict perioperative complications. Furthermore, evidence is scarce on body composition assessment with regard to BA.

Therefore, we performed an analysis of our previously described cohort of patients with SSI and the subcohort of patients with BA^6^ in order to assess possible implications of body composition on the risk of BA development with the aim to simplify risk stratification in clinical routine.

## Methods

The study was approved by the Ethics Review Board of the University of Leipzig under the reference “419/18-ek” and was retrospectively registered in the German register for clinical trials (DRKS, DRKS00019058, 19th December 2019). Due to the retrospective nature of the study the need for informed consent was waived by the Ethics Review Board of the University of Leipzig. The research was carried out in accordance with local legislation and the Declaration of Helsinki.

The exact selection strategy of the cohort has been described previously^6^. In brief, we retrospectively assembled a cohort of patients with SSI from our institution (2015–2018) from their prospectively collected data in the patient chart and assessed their risk factors for the development of BA via descriptive statistics and binary logistic regression model.

The abdominal wall closure was performed in a mass suturing technique with a running suture of PDS loop of the suture strength 1 (Ethicon, Johnson&Johnson, Norderstedt, Germany). No prophylactic meshes, neither absorbable nor permanent have been used at that time.

The BA group was reconsidered in this study as interventional cohort (IC). To establish a control cohort (CC), we randomly selected 35 patients out of the previously established wound register cohort from an anonymized datasheet with a midline laparotomy, that only had developed an SSI, but no BA. We chose the patients with median laparotomy, as this kind of incision is known to cause more wound complications opposed to other incisions. Of those 35 patients 32 had adequate imaging for evaluation to receive an approximate case to control ratio of 3:1. The ratio of 3:1 was obtained from power calculations (clincalc.com) comparing preliminary measurements of visceral obesity (alpha error 0.05; power 0.8) with the aim to use the whole burst abdomen cohort with available CT imaging.

CT images were obtained during clinical routine work-up with suspicion of inflammation or tumor staging. CT was performed on a 128-slice CT scanner (Ingenuity 128, Philips, Hamburg, Germany). Intravenous administration of an iodine-based contrast medium (90 mL Imeron 400 MCT, Bracco Imaging Germany GmbH, Konstanz, Germany) was given at a rate of 4.0 mL/s via a peripheral venous line. Automatic bolus tracking was performed in the descending aorta with a trigger of 100 Hounsfield units (HU). CT was performed with at least a portal-venous phase in every case. Typical imaging parameters were: 100 kVp; 125 mAs; slice thickness, 1 mm; pitch, 0.9.

**Figure 1:**
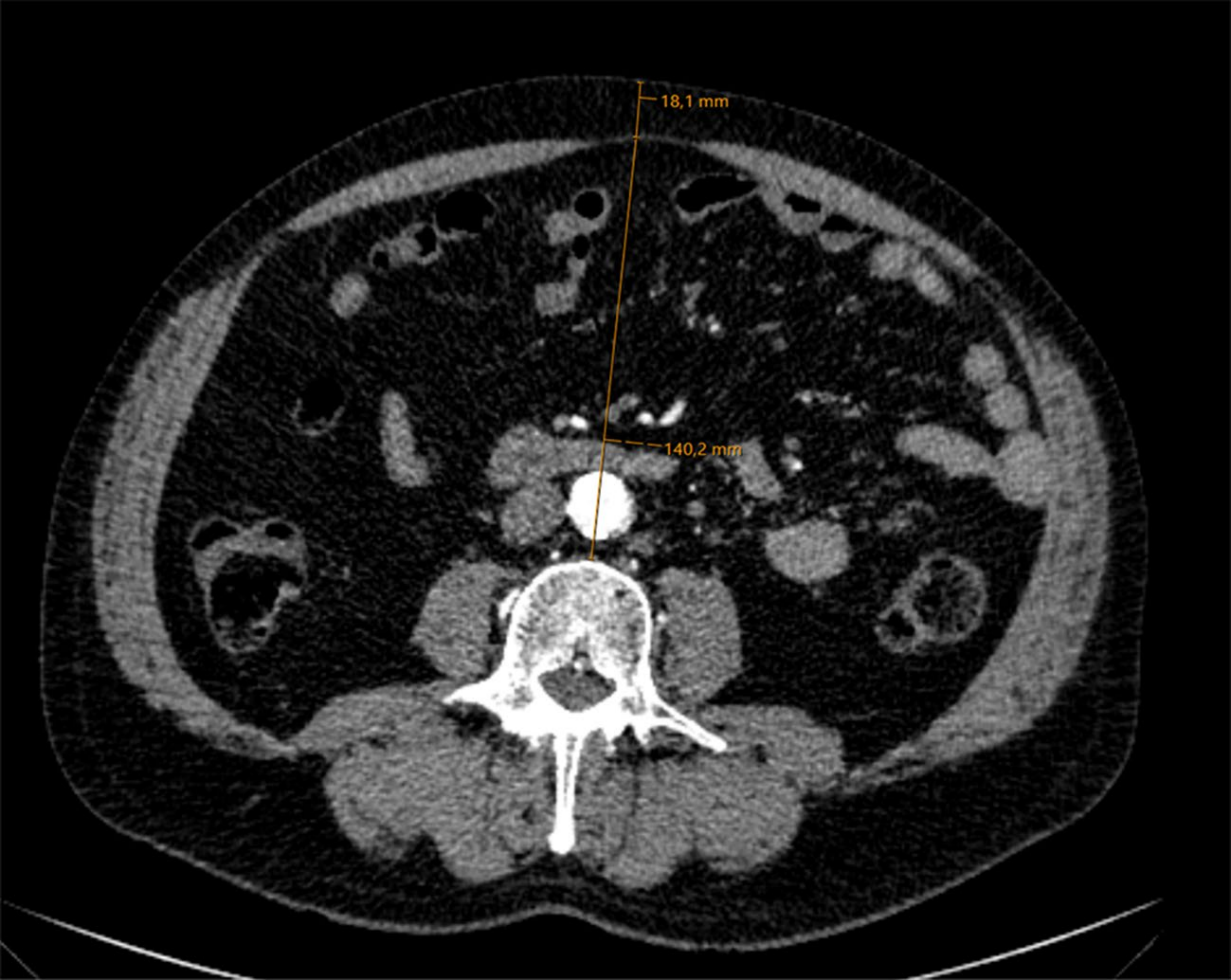
Contrast enhanced CT scan showing the measurement (orange line) of the visceral obesity from the ventral L3 vertebra to the linea alba and subcutaneous obesity from the linea alba to the skin.

The fat tissue parameters were measured in one axial CT slice at the level of the L3 vertebra, as at this level mostly mesenteric fat is depicted on CT slices and no colonic distension would influence the measurements. Visceral obesity (VO) was determined from the ventral aspect of the vertebra to the rectus sheath in the anterior–posterior direction (AP). Subcutaneous obesity (SCO) was represented by the distance between rectus sheath and skin level. Figure 1 shows an example of the typical measurement. Combined Obesity (CBO) was the addition of both distances. Additionally, the ratio between both values was calculated (visceral to subcutaneous ratio = VSR) in order to generate an expression for fat tissue distribution and in response to Kim et al.^8^.

For descriptive statistics we calculated mean (± standard deviation) for continuous variables and relative frequencies for dichotomous variables.

The univariate analysis included the use of unpaired t-test for continuous variables and the X^2^-test or the exact Fisher test for dichotomous variables.

For correlations of continuous variables, the Pearson correlation coefficient was used after testing for normal distribution. For the multivariate regression analysis, the binary logistic regression model with backward stepwise inclusion was chosen.

The significance level was set at *p* = 0.05.

The statistical analysis was performed using SPSS 27 (IBM statistics, Ehningen, Germany).

### Ethical approval

The study was approved by the Ethics Review Board of the University of Leipzig under the reference “419/18-ek” and was retrospectively registered in the German register for clinical trials (DRKS, DRKS00019058, 19th December 2019). Due to the retrospective nature of the study the need for informed consent was waived by the ethics review board.

## Results

### Patient cohort

A total of 494 patients were included in the wound register and 111 patients developed a BA. Of these, 92 had abdominal CT images acquired before the initial surgery and were available for data analysis (IC). Furthermore, a CC of 32 patients with midline laparotomies and subsequent SSI was established. The main patient characteristics for the complete cohort (OC), IC and CC can be deduced from Table 1.

**Table 1:** Patient characteristics of the complete cohort, burst abdomen group (BA) and control group.

	All (n = 124)	Burst abdomen (n = 92)	Control (n = 32)	*p* value (< 0.05)
Sex male /female	81 (65.3)/43 (34.7)	66 (71.7)/ 26 (28.2)	15 (46.9)/17 (53.1)	0.017*
Age	63.98 (± 14.5)	63.61 (± 13.83)	65.04 (± 16.47)	0.633
Median laparotomy	65.3 (81)	53.3 (49)	100 (32)	
Intestinal resection	36.3 (45)	42.4 (39)	18.8 (6)	0.019*
Emergency	50.8 (63)	53.3 (49)	43.8(14)	0.414
Liver cirrhosis	14.5 (18)	18.5 (17	3.1 (1)	0.04*
Delirium	9.7 (12)	10.9 (10)	6.3 (2)	0.729
Hypertension	65.3 (81)	66.3 (61)	62.5 (20)	0.830
Peripheral artery disease	4 (5)	3.3 (3)	6.3 (2)	0.603
Congestive heart failure/coronary artery disease	29.0 (36)	26.1 (24)	37.5 (12)	0.260
Diabetes mellitus	16.9 (21)	18.5 (17)	12.5 (4)	0.587
Dementia	3.2 (4)	2.2 (2)	6.3 (2)	0.274
Malignancy	56.5 (70)	57.6 (53)	53.1 (17)	0.684
Chemotherapy	23.4 (29)	23.9 (22)	21.9 (7)	1
Immunosuppression	18.5 (23)	23.9 (22)	3.1 (1)	0.008**
Chronic inflammatory disease	16.1 (20)	18.5 (17)	9.4 (3)	0.277
Total length of stay in days	34.73 (± 19.35)	38.8 (± 19.71)	23.00 (± 12.43)	< 0.001

All values are given as percentage (absolute number) or as mean (± standard deviation). The given *p* values relate to the comparison of BA group and control group with significance levels set at *p* < 0.05. Significant *p* values are highlighted using * and values of *p* < 0.01 with **.

The majority of patients in the IC were male (71.7%). As surgical approach, most of the laparotomies were performed via median laparotomy (53.3%), transverse abdominal with median epigastric laparotomy (23.9%) or transverse abdominal laparotomy (14.1%).

The CC exhibited statistically equal characteristics except for sex and frequency of midline incision as the IC in the univariate analysis (Table 1). Therefore, the randomly selected control group showed statistical equality compared to the intervention group regarding potential risk factors for BA. The sex difference was similar to the previous analysis^6^ and thus not considered as bias.

To exclude potential bias by the difference in laparotomies used, especially regarding the transverse abdominal incision, during the further analysis the obesity parameters as well as the multivariate regressions were calculated with and without inclusion of patients via transverse incisions. The values did not differ significantly (data not shown). Thus, a bias by inclusion of the transverse abdominal incisions could also be excluded and the results presented further are of the whole cohort.

### CT-derived parameters in overall cohort

CT-derived parameters revealed significantly different distances of the VO and CBO. The SCO did not reach significance levels (*p* = 0.126). Nonetheless, SCO was about 20% higher in the control group than in the IC (2.11 cm vs. 1.75 cm).

To elucidate the relation between obesity in different body compartments and overall obesity, the correlation of the parameters with the BMI as standard parameter for the definition of obesity was used. We could find good correlation between VO and BMI (r = 0.452; *p* < 0.001), and CBO (0.572; *p* < 0.001), moderate correlation of the SCO with BMI (0.373; < 0.001). Hence, combined obesity correlated well with BMI (0.572; *p* < 0.001). In contrast, no correlation was observed between VSR and BMI (−0.007; *p* = 0.943). SCO and VO were inversely correlated (r = −0.189; *p* = 0.035).

### CT-derived parameters comparing both cohorts

The fat distribution parameters were significantly higher in the IC for visceral obesity than in CC (12.72 cm (± 3.05 cm) vs. 9.91 cm (± 2.72 cm), *p* < 0.001 respectively) and subsequently overall obesity (14.49 cm (± 3.61 cm) vs. 12.06 cm (± 3.05 cm), *p* < 0.001). The VSR was 1.78-fold higher in the IC than in controls (10.89 (± 12.18) vs. 6.12 (± 4.24); *p* = 0.002) (Table 2).

**Table 2:** CT-derived parameters of abdominal obesity of the complete cohort and the subgroups.

	Overall	Burst abdomen	Control	*p* value
BMI	26.44 (± 5.78)	26.74 (± 5.74)	25.5(± 5.92)	0.336
Visceral obesity	11.99 (± 3.67)	12.72 (± 3.68)	9.81 (± 2.77)	< 0.001**
Subcutaneous obesity	1.84 (± 1.13)	1.75 (± 1.15)	2.11 (± 1.04)	0.126
Combined obesity	13.84 (± 3.64)	14.48 (± 3.61)	12.00 (± 3.08)	< 0.001**
VSR	9.63 (± 10.83)	10.89 (± 12.18)	6.22 (± 4.27)	0.002**

BMI Body mass index in kg/m^2^, VSR visceral to subcutaneous ratio. The given *p* values relate to the comparison of BA group and control group using the X^2^-test with significance levels set at *p* < 0.05. Significant *p* values are highlighted using * and values of *p* < 0.01 with **.

### Multivariate regression of risk factors for BA

In the previous study, the main risk factors for BA in the IC were liver cirrhosis, intestinal resection, emergency surgery, post-operative delirium and sex. In the present analysis, also immunosuppression was significantly overrepresented in the IC compared to controls. Therefore, those parameters as well as the CT-derived parameters VO, SCO, CBO and VSR were added in the multivariate regression analysis. In the backward stepwise regression model, all previously identified parameters except for immunosuppressants (OR 11.877, 95% CI 1.476–95.579, *p* = 0.02) and intestinal resection (OR 2.654, 95% CI 0.918–7.671; *p* = 0.071) were excluded from the regression model although the latter did not reach significance. Of the obesity parameters, solely VO remained in the equation as risk factor (OR 1.257; 95% CI 1.084–1.459; *p* = 0.003). During the calculation process of the multivariate analysis, the obesity parameters CBO and SCO were the first parameters to be excluded.

## Discussion

In this study we present the CT-derived visceral obesity as one major predictive risk factor for the development of post-operative abdominal fascial dehiscence.

Previously, many clinical parameters and patient specific characteristics have been assessed to predict post-operative abdominal wall dehiscence and subsequent scores have been established^10,11^. These scores could generate decent predictive power, but the main disadvantage was the large number of parameters.

In general, risk scores that predict surgical complications may be of clinical interest in the era of prehabilitation as they would help identify patients in whom prehabilitation would be worthwhile. Although, even scores with few parameters had to be simplified for daily routine (i.e. SOFA^12^), so scores with ten parameters will not be used. Therefore, BA prediction by inclusion of radiologic parameters could be useful.

Neither the scores nor our analysis could show BMI as risk factor for BA although obesity is a known risk-factor for SSI. The use of BMI as surrogate for obesity was less predictive for SSI than subcutaneous fat layer, either by determination intraoperatively^13^ or by CT scan^7^. Although the World Health Organization defines obesity according to BMI, ethnic origin relativizes strict BMI definitions and several studies suggest lower BMI borders for obesity related diseases^14^. Our results are in line with other reports that suggest negative traits of VO assessed by CT scans: Coronary heart disease or combined heart disease^15^. This study mostly links the VO to its negative endocrine functions which increases the incidence of diabetes, non-alcoholic fatty liver disease, cancer or chronic kidney failure^14^. Some metabolic disorders have negative influence on wound healing, such as diabetes mellitus. In the context of BA, rather mechanical properties of intraabdominal adipose tissue mass seem relevant for the prediction of BA development.

Therefore, it seems necessary to evaluate other factors than BMI and their potential risk for BA. Nonetheless, our CC showed higher values of SCO than IC. This coincides with the inverse correlation of VO and SCO: i.e., patients with more SCO are more likely to have less intraabdominal fat. Thus, subcutaneous fat is predictive for subcutaneous impaired wound healing, but increased VO increases the risk of BA because of increased intraabdominal pressure and subsequent tension on the abdominal fascial layers.

Evidence is scarce on the predictive power of CT-derived obesity parameters in the context of BA. Only Nattenmüller et al.^16^ stated an increased risk of BA in patients with increased adipose tissue mass in patients with rectal cancer surgery, but only 3.4% (n = 10) developed BA which is a far smaller sample size than ours. Recently, Kvist et al.^17^ studied the importance of CT-derived values for subcutaneous obesity and could not find an association. This is in line with our findings, that did not promote subcuteanous but visceral obesity as a predictive parameter for postoperative BA in a large cohort.

The methods used to assess patients’ obesity in CT scan vary across studies. A widely used method is the quantification of the area of either subcutaneous or intraabdominal fat in axial slices of contrast enhanced CT scans at the level of the third lumbar vertebra (L3)^8,18^ or the umbilicus^9^. Others have quantified the distance between the anterior aspect of vertebral body to the linea alba and the skin^7^. All studies emphasized associations between obesity parameters and either SSI^7–9^ or other obesity related diseases. On imaging that does not include L3, determination of intraabdominal adipose tissue at the L1/L2 -level also allows reliable measurements^19^. We subsequently chose measurement at level L3.

We chose a simplified approach analogous to Lee et al.^7^ with quantification of the distance between the L3 and linea alba or the skin, respectively. This was also in order to provide a simple tool for the stratification of a patient’s risk of BA during clinical routine as the measurements of adipose tissue area usually require software or a radiologist with special skills. To conclude, our data obtained from those measurements clearly could link VO with the risk of suffering from postoperative BA.

## Limitations

Our study has several limitations which are based on its retrospective nature, with some missing image data sets. Furthermore, our cohort consisted of patients with postoperative SSI. So, our data discriminates between patients with SSI and BA but not between patients with uneventful recovery.

## Conclusion

We show that CT-derived visceral obesity was significantly associated with an increased risk of BA following abdominal surgery. Our findings will help to assess patients’ individual risk of BA with subsequent application of surgical prevention strategies. Further large-scale data will provide additional knowledge on risk stratification of patients.

## Data Availability

The data set used for this study is available from the corresponding author upon reasonable request.
